# Thyroid hormone disorder and the heart: The role of cardiolipin in calcium handling

**DOI:** 10.1113/EP090817

**Published:** 2023-01-18

**Authors:** Valentina D'Angelo, Candela Martinez, Noelia Arreche, Ana María Balaszczuk, María del Carmen Fernández, Juan Ignacio Burgos, Martin Vila Petroff, Andrea Fellet

**Affiliations:** ^1^ Cátedra de Fisiología, Facultad de Farmacia y Bioquímica, IQUIMEFA‐CONICET Ciudad Autónoma de Buenos Aires Universidad de Buenos Aires Buenos Aires Argentina; ^2^ Cátedra de Biología Celular y Molecular Facultad de Farmacia y Bioquímica IQUIFIB‐CONICET Ciudad Autónoma de Buenos Aires Universidad de Buenos Aires Buenos Aires Argentina; ^3^ Centro de Investigaciones Cardiovasculares Horacio Cingolani. Facultad de Ciencias Médicas Universidad Nacional de La Plata CONICET La Plata Argentina

**Keywords:** calcium, cardiolipin, heart, thyroid hormone

## Abstract

The objective of this study was to evaluate whether alterations in thyroid status affect (1) haemodynamic parameters and echocardiographic measurements in the rat postnatal heart, and (2) calcium handling, contractility, relaxation and cardiolipin content in isolated rat cardiomyocytes. Sprague–Dawley rats aged 2 months treated with T_3_ (hyperthyroid, 20 μg/100 g body weight) or 0.02% methimazole (hypothyroid, w/v) for 28 days. Heart function was evaluated by echocardiography. Measurements of mean arterial pressure (MAP), heart rate, Ca^2+^ transients, cardiomyocyte shortening, number of spontaneous contractions per minute and cardiolipin (CL) content were performed. Thyroid disorders were associated with changes in pacemaker activity without modifications of MAP. Thyroid disorder induced changes in left ventricular diameter which were correlated with modifications of cardiac contractility (altered cell shortening and sarcoplasmic reticulum Ca^2+^ content). Endocrine disorders altered cardiomyocyte relaxation (reduction in the time to 50% re‐lengthening and the time to 50% Ca^2+^ decay). Thyroid disorder increased the number of spontaneous contractions per minute (an index of pro‐arrhythmogenic behaviour). CL content was increased only in hypothyroid rats. Changes in CL content, CL composition and CL–protein interaction in mitochondria from hypothyroid animals are responsible for alterations of contractile and relaxation cardiac function. This mechanism may be not be involved in T_3_‐treated rats. Maintenance of euthyroidism is of crucial importance to preserve cardiac performance. An imbalance in relation to phospholipids of the mitochondrial membrane such as CL is related to defects in mitochondrial function. T_3_‐dependent CL signals contribute to the maintenance of mitochondrial homeostasis and involve Ca^2+^ handling, this pathway being more important in hypothyroidism.

## Abstract (highlights)

- **What is the central question of this study?**
  Do alterations in thyroid status affect haemodynamic parameters and echocardiographic measurements in the rat postnatal heart, and calcium handling, contractility, relaxation and cardiolipin content in isolated rat cardiomyocytes?
- **What is the main finding and its importance?**
  An imbalance in phospholipids of the mitochondrial membrane such as cardiolipin is related to defects in mitochondrial function. T_3_‐dependent cardiolipin signals contribute to the maintenance of mitochondrial homeostasis and involve Ca^2+^ handling, this pathway being more important in hypothyroidism.

## 1 INTRODUCTION

Previous evidence reported by our laboratory suggests that the nitric oxide (NO) pathway is one of the mechanisms involved in heart homeostasis during disorders of thyroid function (Fellet et al., 2008; Rodriguez et al., 2015; Sarati et al., 2012). However, knowing also that thyroid hormones affect the composition of the mitochondrial membrane (Chicco & Sparagna, 2007), other factors such cardiolipin (CL) content could be implicated in altered cardiac function. CL is one of the principal phospholipids in the mammalian heart, comprising as much as 15–20% of the entire phospholipid phosphorus mass of that organ. It is localized primarily in the inner mitochondrial membrane and appears to be crucial for the optimal function of numerous membrane proteins, playing an important role in energy metabolism and production of energy for the heart to beat (Paradies et al., 2019). Its synthesis occurs via the cytidine‐5′‐diphosphate‐1,2‐diacyl‐*sn*‐glycerol pathway and is mainly regulated by the energy status of the heart. In this context, thyroid hormones (TH) may regulate CL biosynthesis. Moreover, it has been reported that inner mitochondrial membrane cardiolipin levels affect calcium handling by regulating stability and abundance of the mitochondrial calcium uniporter (MCU) (Ghosh et al., 2020), among other transporters. Thus, an interesting issue arises as to whether heart mitochondrial CL content is associated with altered myocardial calcium dynamics that occur due to disorders of thyroid status. It is well known that THs are necessary for optimal cardiac myocyte relaxation and contraction, controlling intracellular Ca^2+^ within the myocyte, the sarco/endoplasmic reticulum Ca^2+^‐ATPase (SERCA), phospholamban and MCU, among others (Vargas‐Uricoechea & Sierra‐Torres, 2014). Therefore, it was hypothesized that an increase of mitochondrial CL content in postnatal heart with thyroid disorder would enhance cytoplasmic Ca^2+^ concentrations modulating myocardial function. So the purpose of this study was to evaluate whether alterations in thyroid status affect (1) haemodynamic parameters and echocardiographic measurements in the rat postnatal heart, and (2) calcium handling, contractility, relaxation and CL content in isolated rat cardiomyocytes.

## 2 METHODS

### 2.1 Ethical approval

All protocols were performed according to the guidelines recommended by the Institutional Committee for the Care and Use of Laboratory Animals (CICUAL) of the School of Pharmacy and Biochemistry, University of Buenos Aires (EXP‐FYB No. 0054570/2015).

### 2.2 Animals

Pregnant Sprague–Dawley rats were obtained from the breeding laboratories of the School of Pharmacy and Biochemistry, University of Buenos Aires, Argentina. Rats were housed one animal per cage under controlled humidity and temperature conditions, with an automatic 12‐h light–dark cycle; they had free access to commercial standard rat chow (Ganave, Buenos Aires, Argentina) and received water ad libitum. Male rat offspring weighing approximately 10 g were used in this study and were randomly assigned to one of the three experimental groups.

### 2.3 Experimental design

Rats were randomly assigned to one of the following three groups. (1) Control rats (Eut, *n* = 15): euthyroid animals that received s.c. injections of 0.9% NaCl (0.1 ml/100 g body weight (BW) every second day for 28 days. (2) Methimazole‐treated rats (Hypo, *n* = 15): animals were rendered hypothyroid after 28 days of treatment with 0.02% methimazole (w/v) in the drinking water (Franco et al., 2006). (3) T_3_‐treated rats (Hyper, *n* = 15): animals received s.c. injections of T_3_ (Sigma Aldrich de Argentina SA, 20 μg/100 g body weight) every second day for 28 days (Heron & Rakusan, 1996).

### 2.4 Determination of treatment efficacy

Serum thyroid‐stimulating hormone (TSH), total triiodothyronine (T_3_) and thyroxin (T_4_) (TSH kit, National Institute of Diabetes and Digestive and Kidney Diseases, National Institutes of Health, Bethesda, MD, USA) were measured in all groups of animals by radioimmunoassay at the beginning and the end of the experimental period (Greeley et al., 1982).

### 2.5 Echocardiographic measurements

After the experimental period (28 days), animals were anaesthetized with urethane (1.0 g/kg, i.p.) and echocardiographic measurements were performed in the left lateral decubitus position. Two‐dimensional directed M‐mode images were obtained using a Sonoscape (A6 Vet) system with a 9–4 MHz transducer. Measurements were taken in the right parasternal short axis plane at the level of the mitral valve leaflets. All determinations were made according to the guidelines of the American Society of Echocardiography (Ogonowski et al., 2016).

### 2.6 Haemodynamic parameters

After echocardiographic measurements, a tracheotomy was performed using polyethylene tubing (4 mm ID, Portex, Argentina SRL). Mean arterial pressure (MAP) was measured through a cannula inserted into the right femoral artery and connected to a pressure transducer (Statham P23 ID, Gould Inst, Cleveland, OH, USA). Measurements were recorded with a polygraph (Physiograph E & M, Houston, TX, USA) during the whole experiment. Heart rate (HR) was determined from the pulsatile pressure signal by beat‐to‐beat conversion with a tachograph amplifier (model S77‐26 tachometer, Coulbourn Instruments, Allentown, PA, USA). The Labtech Notebook program (Laboratory Technology, Wilmington, MD, USA) was used for data acquisition.

### 2.7 Cardiomyocyte isolation

After haemodynamics measurements, animals killed by cervical dislocation and hearts were rapidly excised. Cardiac myocytes were isolated by collagenase‐based enzymatic digestion according to a technique previously described (Sepulveda et al., 2013). After isolation cells were kept in a HEPES solution, containing (in mmol/l): 146.2 NaCl, 4.7 KCl, 1.8 CaCl_2_, 10.0 HEPES, 0.4 NaH_2_PO_4_, 1.1 MgCl_2_, 10 glucoses (pH adjusted to 7.4 with NaOH), at room temperature (20–24°C) until use.

#### 2.7.1 Calcium transient and cell shortening measurements

Ca^2+^ transient and cardiomyocyte shortening measurements were performed at room temperature, as previously described (Burgos et al., 2017). Briefly, the cardiomyocytes were placed in a perfusion chamber on the stage of an inverted microscope (Nikon, Tokyo, Japan), continuously superfused with a HEPES solution and stimulated via two platinum electrodes on either side of the bath at 0.5 Hz. Resting cell length and cell shortening were measured using a video‐based motion detector (Ionoptix, Westwood, MA, USA) and stored by software for off‐line analysis. The average values of the inotropic response were calculated after a 10 min stabilization period (control). The time to 50% re‐lengthening was measured as an index of relaxation. For intracellular Ca^2+^ measurements, isolated cardiomyocytes were loaded with 10 μmol/l Fura‐2 AM (Thermo Fisher Scientific, Waltham, MA, USA) for 12 min at room temperature. The ratio of the Fura‐2 fluorescence (510 nm) obtained after exciting the dye at 340 and 380 nm was taken as an index of free cytosolic Ca^2+^. Time to 50% relaxation was used as a parameter of the rate of Ca^2+^ decay.

### 2.8 Cardiolipin analyses

After animal killing, the heart was removed and kept in an ice‐cold Krebs–Ringer bicarbonate buffer containing 5.5 mM glucose. The medium was gassed with 95% O_2_–5% CO_2_. For each phospholipid biosynthesis experiment, heart was sliced (0.5 mm thick) using a Stadie–Riggs microtome. Tissue slices (10 mg wet weight) were collected in 200 μl of ice‐cold 10 mM Tris–HCl buffer, pH 7.4, containing 5.5 mM glucose, 140 mM NaCl, 5 mM KCl, 2 mM MgSO_4_ and 1 mM CaCl_2_. For each experiment, on the other hand, samples of 30 mg of heart (not sliced) were collected in 500 μl of ice‐cold Krebs–Ringer bicarbonate buffer, pH 7.4, containing 5.5 mM glucose. Total lipids were extracted using the Bligh–Dyer method. Phospholipid identification and isolation was made by thin layer chromatography. Finally, CL quantification was performed using Bartlett's technique.

### 2.9 Statistical analysis

Data in tables and figures are mean values ± SD. Data were evaluated with univariate and multivariate approaches for a completely randomized design. For each variable, ANOVA or MANOVA analysis was performed where appropriate. Levene's and Shapiro–Wilk's tests were used to evaluate homogeneity of variances and normality of data, respectively. When normality and homogeneity of variance assumptions were satisfied, the Bonferroni multiple comparison test was run. In the case of non‐homogeneous variances, a multiple comparisons test, such as Tamhane's, was run. To detect association among variables, a correlation analysis was performed, and Pearson's coefficient was calculated. All statistical procedures were performed using the SPSS Statistics version 22.0 (IBM Corp., Armonk, NY, USA); statistical significance was set at *P* < .05.

## 3 RESULTS

Methimazole and T_3_ treatment were effective in inducing thyroid disorder. In addition, body weight values were similar between the three groups of animals. When haemodynamic parameters were evaluated, HR decreased and increased in Hypo and Hyper groups, respectively. Meanwhile, MAP values were similar in the three groups of animals (Table 1).

**TABLE 1:** Thyroid state and body weight.

	Eut	Hypo	Hyper
TSH (ng/ml)	12.55 ± 1,591	38.59 ± 5,495*	7.45 ± 0.347*
T_3_ (ng/dl)	1.162 ± 0,476	0.650 ± 0.139*	1.345 ± 0.008*
T_4_ (ng/ml)	2.355 ± 0.081	1.055 ± 0.097*	4.575 ± 0.949*
BW (g)	347 ± 46	355 ± 46	335 ± 46

Data are mean ± SD; *n* = 15.

^*^ *P* < 0.05 versus Eut. BW, body weight; Eut, euthyroid rats; Hyper, hyperthyroid rats; Hypo, hypothyroid rats; T_3_, triiodothyronine; T_4_, thyroxine.

**FIGURE 1:**
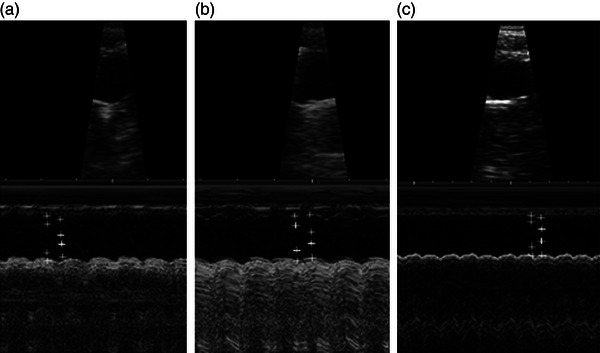
Representative images of left ventricular M‐mode echocardiographic tracing from euthyroid (a), hypothyroid (b) and hyperthyroid (c) rats.

Echocardiographic measurements reveal that left ventricular internal diameter during systole and diastole increased in Hypo and decreased in Hyper rats. Furthermore, anterior wall thickness and posterior wall thickness decreased only in hypothyroidism and did not change in the Hyper group. Similar changes were observed in ejection fraction and fractional shortening (Table 2). Figure 1 shows representative images of M‐mode echocardiographic tracing for each experimental group.

**TABLE 2:** Biological variables.

	Eut	Hypo	Hyper
HR (bpm)	355 ± 43	225 ± 58*	435 ± 50*
MAP (mm Hg)	84 ± 12	73 ± 12	77 ± 19
LVIDd (mm)	5.38 ± 0.46	6.02 ± 0.46*	4.3 ± 0.46*
LVIDs (mm)	2.70 ± 0.15	3.12 ± 0.39*	2.10 ± 0.27*
AWTd (mm)	1.50 ± 0.08	1.35 ± 0.04*	1.55 ± 0.19
AWTs (mm)	2.50 ± 0.12	2.00 ± 0.08*	2.60 ± 0.19
PWTd (mm)	2.10 ± 0.66	1.50 ± 0.39*	2.03 ± 0.58
PWTs (mm)	2.85 ± 0.19	2.33 ± 0.39*	3.01 ± 0.19
EF (%)	87 ± 12	80 ± 4*	90 ± 12
FS (%)	55 ± 12	45 ± 8*	57 ± 4

Data are mean ± SD; *n* = 15.

^*^ *P* < 0.05 versus Eut. AWT, anterior wall thickness; d, diastole; EF, ejection fraction; Eut, euthyroid rats; FS, fractional shortening; HR, heart rate; Hyper, hyperthyroid rats; Hypo, hypothyroid rats; LVID, left ventricle internal diameter; MAP, mean arterial pressure; PWT, posterior wall thickness; s, systole.

**FIGURE 2:**
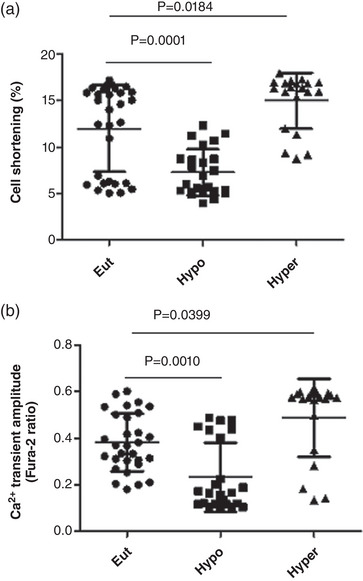
Changes in cell shortening (a) and Ca^2+^ transient amplitude (b) in euthyroid (Eut), hypothyroid (Hypo) and hyperthyroid (Hyper) rats. Data are means ± SD; *n* = 7/group.

Figure 2 shows that contractility was decreased and increased in Hypo and Hyper animals, respectively. These contractility alterations induced by thyroid disorder were associated with altered cell shortening and sarcoplasmic reticulum Ca^2+^ content (Figure 2a, b).

**FIGURE 3:**
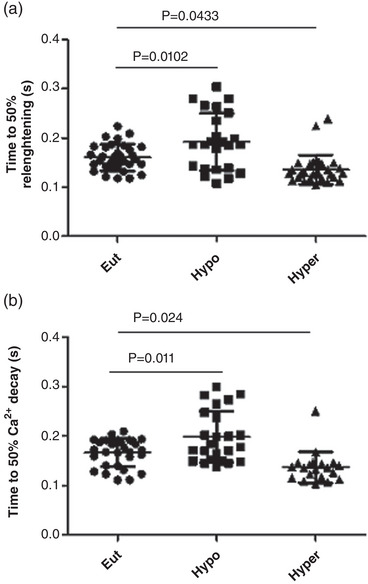
Time to 50% re‐lengthening (a) and time to 50% Ca^2+^decay (b) in in euthyroid (Eut), hypothyroid (Hypo) and hyperthyroid (Hyper) rats. Data are means ± SD; *n* = 7/group.

Cardiomyocyte relaxation as revealed by a reduction in the time to 50% re‐lengthening was also changed in both endocrinal alterations. This parameter was increased and decreased in Hypo and Hyper groups, respectively. Additionally, hormonal deficiencies also increased the time to 50% Ca^2+^ decay whereas Hyper animals showed a decrease of this time (Figure 3a, b).

**FIGURE 4:**
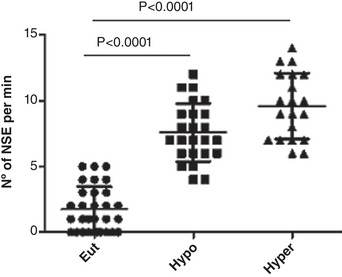
Spontaneous contractions per minute (NSE) in euthyroid (Eut), hypothyroid (Hypo) and hyperthyroid (Hyper) rats. Data are means ± SD; *n* = 7/group.

**FIGURE 5:**
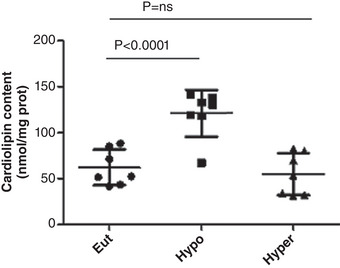
Cardiolipin content in in euthyroid (Eut), hypothyroid (Hypo) and hyperthyroid (Hyper) rats. Data are means ± SD; *n* = 7/group.

Figure 4 shows that the number of spontaneous contractions per minute, used as an index of pro‐arrhythmogenic behaviour, was significantly increased in the cardiomyocytes of the Hypo and Hyper rats. CL content was increased in hypothyroid rats, but was unchanged in hyperthyroidism (Figure 5).

## 4 DISCUSSION

This study was conducted to evaluate the potential molecular mechanisms involved in the alterations of contractility and heart relaxation in postnatal thyroid disorder. Our findings suggest that maintenance of euthyroidism is fundamental for the preservation of cardiac performance. The relationship between thyroid function and cardiac disease is complex. Our results showed that methimazole and T_3_ treatment were effective in establishing thyroid disorder. Thyroid postnatal alterations were reflected by hormonal serum measurements.

It is well known that hyper‐ and hypothyroidism are associated with functional and structural alterations of the heart, as well as alterations of peripheral vascular resistance and blood pressure (Osuna et al., 2017; Sepulveda et al., 2013). In this study, MAP values were similar between the three experimental groups. This finding could be explained considering that changes of diastolic pressure were similar in magnitude to those induced by systolic pressure despite having very different HR values in the two thyroid states. This discrepancy with other research might be due to the different duration and degree of hypo‐ and hyperthyroidism developed in our experimental conditions. It is well known that thyroid state has a specific influence on the blood vessel wall.

Hypothyroidism impairs cardiovascular health and contributes to endothelial dysfunction with reduced vasodilatation, while hyperthyroidism has an opposite effect (Chicco & Sparagna, 2007). When we evaluated cardiovascular function, the echocardiographic data showed that hypothyroid animals have decreased fractional shortening and ejection fraction and increased left ventricle internal diameter. Hyperthyroid animals, by contrast, showed a decrease in the diameter of the ventricle without changes in cardiac contractility in our experimental model (Sarati et al., 2012). These findings disagree with those of Weltman et al. who showed that hyperthyroidism increases the risk of myocardial remodelling such as chamber dilatation and heart failure and low cardiac output (Weltman et al., 2012). Other authors indicated that hyperthyroidism was associated with increased heart rate and output and decreased total peripheral resistance (Guerri et al., 2019). In our experimental model, only hypothyroid animals had decreased left ventricular contractile function. The latter is in accordance with several studies which showed that hypothyroidism induces a decrease in cardiac output, HR and diastolic function and a rise in peripheral vascular resistance (Vargas‐Uricochea et al., 2014). This led us to investigate the mechanism by which TH could affect cardiac contractility.

Ca^2+^ transient and cardiomyocyte shortening measurements in isolated cardiomyocytes showed a decrease and increase in hypo‐ and hyperthyroidism, respectively. Cardiomyocyte relaxation time increased and decreased in the Hypo and Hyper groups, respectively. It is well known that THs have extensive effects on the cardiac muscle, where essential proteins tightly regulate intracellular Ca^2+^ within the myocyte, such as SERCA, phospholamban and MCU, among others (Collin et al., 2005; Pantos et al., 2007). Cardiac contractility depends on SERCA2 and myosin heavy chains α and β (Paavola et al., 2020). Several authors showed that T_3_ favourably regulates SERCA2 while negatively regulating its inhibitory equivalent, phospholamban (Ketzer et al., 2009). It is well demonstrated that THs are necessary for optimal myocyte relaxation and contraction of cardiac myocytes (Klein, 1990). Numerous studies have demonstrated that differences in TH levels affect mitochondrial Ca^2+^ transport in cardiac mitochondria. Through variations in the expression of two uniplex subunits, MICU1 and MCU2, mitochondrial Ca^2+^ uptake occurs through a highly selective Ca^2+^ channel known as the MCU complex or uniplex, located on the inner mitochondrial membrane (Alevriadou et al., 2021; Bisbach et al., 2020).

In addition, the results showed that the number of spontaneous contractions per minute was significantly increased in the cardiomyocytes of the Hypo and Hyper rats. It is well known that TH also influences pacemaker activity. T_3_ has an effect on sodium pump channels, Na^+^/K^+^ permeability and ion current in sinoatrial node (Renaudon et al., 2000). However, our experiments reflect direct actions of THs on cardiomyocyte membranes. Moreover, this could be partially attributed to an increased Ca^2+^ leaking from the mitochondria related to a phospholipid imbalance. In this context, CL acyl composition might also affect calcium handling protein. This effect was not fully evaluated and is a promising area for further studies. In this context, it is important to note that in a previous study from our laboratory, we showed that heart mitochondrial function is altered during thyroid disorder. We demonstrated that decreased TH level would probably be a hormonal environment that promotes changes in mitochondrial NO bioavailability modulating oxygen consumption and cell respiration. Alterations of complex I activity could mediate these effects (Ogonowski et al., 2018). We suggest that these mitochondrial alterations associated with thyroid disorder may be responsible for cardiovascular alterations. On the other hand, in the last decade increased evidence has been obtained on the importance of CL in heart mitochondrial function. The anionic lipid CL is linked to numerous mitochondrial inner membrane proteins, particularly those of the respiratory chain, and is necessary for optimal mitochondrial function (Paradies et al., 2019). Studies conducted in vitro revealed that CL interacts with a variety of mitochondrial proteins and is necessary for multiple enzymes in the mitochondrial respiratory chain to function at their peak levels, such as cytochrome *c* oxidase activity, NADH dehydrogenase (complex I), ubiquinol: cytochrome *c* oxidoreductase (complex III), cytochrome *c* oxidase (complex IV) and ATP synthase (complex V), among others (Szeto & Liu, 2018). However, little is known regarding the effects of CL on heart calcium handling. In the heart, calcium regulates sarcomere contraction and relaxation and is crucial in coupling mitochondrial metabolism and energy‐intensive myofilament activities. During excitation, MCU transports Ca^2+^ from the cytosol into mitochondria, activating Krebs cycle flux under the effects of increased workload. It is crucial to bear in mind that MCU requires CL for optimal activity (Yeung & Prakriya, 2020).

On the other hand, besides their function in energy conversion, mitochondria participate in multiple metabolic pathways, such as the urea cycle, the metabolism of amino acids and lipids, and the biogenesis of haem and iron sulfur clusters (Smith et al., 2018). Numerous signalling pathways, including innate immunological responses, calcium signalling and programmed cell death, among others, depend on mitochondrial dynamics. Most of the key metabolic enzymes of the mitochondria are embedded in the inner membrane. CL is the characteristic lipid of the inner membrane, playing a key part in the majority of mitochondrial metabolic processes (Paradies et al., 2019; Szeto & Liu, 2018). Here, we will discuss how CL is involved in many essential mitochondrial functions including calcium handling during thyroid disorder. Therefore, Ca^2+^ plays a leading role in adapting mitochondrial metabolism to increased energy demands during accelerated cardiac workload.

Defects in the biosynthesis and remodelling of CL have a significant impact on mitochondrial activity, specifically affecting tissues with a large energy input from mitochondria, such as the heart (Chicco & Sparagna, 2007). Sengers disease, Barth syndrome, and dilated cardiomyopathy with ataxia are illnesses having a direct connection to CL biosynthesis and remodelling (Vargas‐Uricochea et al., 2014). The results of the present study provide a starting point for investigating the molecular mechanism and pathophysiology of mitochondrial disease associated with thyroid disorder. Duncan et al. (2018) suggested that CL is required for optimal functioning of the mitochondrial carriers. In this study we showed that CL content was increased in Hypo rats, without changes in hyperthyroidism. Hypothyroidism induced a rise in CL levels, altering mitochondrial function. This could reduce MCU activity and calcium import within mitochondria and alters heart function (lower fractional shortening and atrial fibrillation). Other cardiac alterations, such as ischaemia–reperfusion injury, diabetic cardiomyopathy and the ageing heart, are also influenced by changes in CL levels (Wasmus & Dudek, 2020). Furthermore, in the Hyper rats where there are no changes in CL content but there are alterations in contractility and the Ca^2+^ transient, these alterations would be independent of CL. Thus, different molecular pathways would affect heart contractile activity during thyroid disorder. Our findings suggest that heart mitochondrial CL content would be mainly involved in hypothyroid animals. The results of the study are contrary to those of several authors who show that decreased CL content is associated with different pathophysiological conditions (Chistiakov et al., 2018). However, our results could be explained considering that when the mitochondria are subjected to stress conditions, it has been reported that cardiolipin translocates to the outer membrane serving as a binding site in many cellular signalling events related to mitophagy and/or the apoptosis process (Kalanxhi & Wallace, 2007). This latter could explain the decreased cardiac function observed in our experimental model. On the other hand, it is well documented that both hypothyroidism and hyperthyroidism can predispose to ventricular arrhythmia and other major adverse cardiovascular events. Several studies showed that between 10% and 15% of hyperthyroid patients develop atrial fibrillation (Higa et al., 2021). However, the impact of hypothyroidism on atrial fibrillation remains unclear. There are a few case reports that have suggested that hypothyroidism induces atrial fibrillation. Our findings are in concordance with those of Li et al. (2019) who demonstrated that although hypothyroidism and hyperthyroidism increase susceptibility to atrial fibrillation, they have different effects on atrial electrophysiological parameters.

It is important to note that thyroid disease is a complex, multifactorial syndrome involving cardiovascular alterations at the molecular, cellular and organ levels where mitochondrial respiratory dysfunction may also play an important role in the pathogenesis and progression of cardiovascular failure. Changes in CL content, CL composition and CL–protein interaction in mitochondria from hypothyroid rats have been responsible for alterations of contractile and relaxation cardiac function (Paradies et al., 1993, 2014, 2019). This mechanism may not be involved in T_3_‐treated rats. The findings in the present study show that maintenance of euthyroidism is of crucial importance to preserve cardiac performance. An imbalance in relation to phospholipids of the mitochondrial membrane such as CL is related to defects in mitochondrial function. T_3_‐dependent CL signals contribute to the maintenance of mitochondrial homeostasis and are involved in Ca^2+^ handling, this pathway being more important in hypothyroidism.

### 4.1 Study limitations

The real cause of the heart dysfunction cannot be determined with our experimental protocol. However, the obtained results led us to infer a causal relationship between heart mitochondrial CL content, Ca^2+^ handling and thyroid status. Although, it is well known that mitochondria are essential for heart function (Chistiakov et al., 2018), the role of CL associated with cardiac alterations induced by hypo‐ or hyperthyroidism has been little examined.

## AUTHOR CONTRIBUTIONS

All authors have contributed to design of the work, acquisition, analysis, or interpretation of data. D'Angelo, Vila Petroff, Balaszczuk and Fellet revised the work critically for important intellectual content. All authors approved the final version of the manuscript and agreed to be accountable for all aspects of the work in ensuring that questions related to the accuracy or integrity of any part of the work are appropriately investigated and resolved. All persons designated as authors qualify for authorship, and all those who qualify for authorship are listed.

## CONFLICT OF INTEREST

Authors declare that there is no conflict of interest that could be perceived as prejudicing the impartiality of the research reported.

## Supporting information

Statistical Summary Document

## Data Availability

The data that support the findings of this study are available in the Supporting information of this article.
