# Supplementary material for: Thyroid hormone disorder and the heart: The role of cardiolipin in calcium handling
Source: Exp Physiol. 2023 Jan 18;108(3):412–9. doi: 10.1113/EP090817 (PMC10103858; doi:10.1113/EP090817)
Supplement: Supplementary file 1 — Statistical Summary Document [file EPH-108-412-s001.docx]

**Manuscript Title:** THYROID HORMONE DISORDER AND HEART: THE ROLE OF CARDIOLIPIN IN CALCIUM HANDLING

**Authors:** Valentina D’Angelo, Candela Martinez, Noelia Arreche, Ana María Balaszczuk, María del Carmen Fernández, Juan Ignacio Burgos, Martin Vila Petroff, Andrea Fellet

**Animal model used, if applicable:** Male Sprague Dawley rats

**Underlying hypothesis:** This investigation tests the hypothesis that an increase of mitochondrial CL content in postnatal heart with thyroid disorder would enhance cytoplasma Ca2+ concentrations modulating myocardial function.

**Definitions of ‘n’:**

n = number of animals of each group

**Statistical summary table:**

| Experimental question number* | Finding/ conclusion | Experimental location/ variable | Mean value | SD | n val. | P** | Units | Data comparisons | Statistical test | Figure/ table | Comments  e.g. observation |
| --- | --- | --- | --- | --- | --- | --- | --- | --- | --- | --- | --- |
| 1. Cell shortening | cell shortening is altered in thyroid disorders | Cardiomyocyte  Eut rats  Hypo rats  Hyper rats | 12,02  7,29  14,99 | 4,66  2,51  3,00 | 30  24  20 | **P<0,0001**  **p=0,0184**  **p<0,0001** | % | Hypo vs Eut  Hyper vs Eut  Hypo vs Hyper | ANOVA  Bonferroni´s multiple comparison test | Fig 2  PanelA | Cell shortening decreased and increased in Hypo and Hyper rats, respectively |
| 2. Ca^2+^ transient amplitude | Sarcoplasmic-reticulum Ca2+ content is altered in thyroid disorders | Cardiomyocyte  Eut rats  Hypo rats  Hyper rats | 0,382  0,233  0,487 | 0,123  0,148  0,167 | 30  24  20 | **p=0,0010**  **p=0,0399**  **p<0,0001** | Fura-2 ratio | Hypo vs Eut  Hyper vs Eut  Hypo vs Hyper | ANOVA  Bonferroni´s multiple comparison test | Fig 2  Panel B | Sarcoplasmic-reticulum Ca2+ content decreased and increased in Hypo and Hyper rats, respectively |
| 3. Time to 50% relengthening | Time to 50% relengthening is altered in thyroid disorders | Cardiomyocyte  Eut rats  Hypo rats  Hyper rats | 0,161  0,193  0,136 | 0,027  0,058  0,030 | 32  24  29 | **p=0,0102**  **P=0,0433**  **p<0,0001** | % | Hypo vs Eut  Hyper vs Eut  Hypo vs Hyper | ANOVA  Bonferroni´s multiple comparison test | Fig 3  Panel A | Time to 50% relengthening Increased and decreased in Hypo and Hyper rats, respectively |
| 4. Time to 50% Ca2+ decay | Time to 50% Ca2+ decay is altered in thyroid disorders | Cardiomyocyte  Eut rats  Hypo rats  Hyper rats | 0,166  0,198  0,136 | 0,028  0,052  0,031 | 30  24  20 | **p=0,011**  **p=0,024**  **p<0,0001** | % | Hypo vs Eut  Hyper vs Eut  Hypo vs Hyper | ANOVA  Bonferroni´s multiple comparison test | Fig 3  Panel  B | Time to 50% Ca2+ decay Increased and decreased in Hypo and Hyper rats, respectively |
| 5. N° of NSE per minute | NSE is altered in thyroid disorders | Cardiomyocyte  Eut rats  Hypo rats  Hyper rats | 1,733  7,583  9,600 | 1,721  2,205  2,501 | 30  24  20 | **p<0,0001**  **p<0,0001**  **p<0,0001** | NSE per minute | Hypo vs Eut  Hyper vs Eut  Hypo vs Hyper | ANOVA  Bonferroni´s multiple comparison test | Fig 4 | NSE increased in Hypo and Hyper rats. |
| 6.Cardiolipin content | Cardiolipin content is altered in thyroid disorders | Heart  Eut rats  Hypo rats  Hyper rats | \|  \| 121 \| 54,8 \| \| --- \| --- \| --- \|   61,9  121,0  54,8 | 19,5  25,3  22,7 | 7  7  7 | **p=ns**  **p=0,0001** | nmol/mg protein | Hypo vs Eut  Hyper vs Eut  Hypo vs Hyper | ANOVA  Bonferroni´s multiple comparison test | Fig 5 | Cardiolipin content increased in Hypo rats |
| 7. TSH | TSH levels are altered in thyroid disorders | Serum  Eut rats  Hypo rats  Hyper rats | 12.55  38.59  7.45 | 1,591  5,495  0,3472 | 15  15  15 | **p<0,0001**  **p=0,0002**  **p<0,0001** | ng/mL | Hypo vs Eut  Hyper vs Eut  Hypo vs Hyper | ANOVA  Bonferroni´s multiple comparison test | Table 1 | TSH serum levels increased and decreased in Hypo and Hyper rats, respectively |
| 8.T_3_ | T_3_ levels are altered in thyroid disorders | Serum  Eut rats  Hypo rats  Hyper rats | 1.162  0.650  1.345 | 0,476  0.139  0.008 | 15  15  15 | **p<0,0001**  **p=0,020**  **p<0,0001** | ng/dL | Hypo vs Eut  Hyper vs Eut  Hypo vs Hyper | ANOVA  Bonferroni´s multiple comparison test | Table 1 | T_3_ serum levels decreased and increased in Hypo and Hyper rats, respectively |
| 9. T_4_ | T_4_ levels are altered in thyroid disorders | Serum  Eut rats  Hypo rats  Hyper rats | 2.355  1.055  4.575 | 0.081  0.097  0.949 | 15  15  15 | **p<0,0001**  **p<0,0001 p<0,0001** | ng/mL | Hypo vs Eut  Hyper vs Eut  Hypo vs Hyper | ANOVA  Bonferroni´s multiple comparison test | Table 1 | T_4_ serum levels decreased and increased in Hypo and Hyper rats, respectively |
| 10.BW | BW is not altered in thyroid disorders | Eut rats  Hypo rats  Hyper rats | 347  355  335 | 46  46  46 | 15  15  15 | p=ns  p=ns  p=ns | g | Hypo vs Eut  Hyper vs Eut  Hypo vs Hyper | ANOVA  Bonferroni´s multiple comparison test | Table 1 | BW values were similar between the three groups of animals |
| 11.HR | HR is altered in thyroid disorders | Eut rats  Hypo rats  Hyper rats | 355  225  435 | 43  58  50 | 15  15  15 | **P<0,0001**  **P<0,0001**  **P<0,0001** | bpm | Hypo vs Eut  Hyper vs Eut  Hypo vs Hyper | ANOVA  Bonferroni´s multiple comparison test | Table 2 | HR decreased and increased in Hypo and Hyper rats, respectively |
| 12. MAP | MAP is altered in thyroid disorders | Eut rats  Hypo rats  Hyper rats | 84  73  77 | 12  12  19 | 15  15  15 | p=ns  p=ns  p=ns | mmHg | Hypo vs Eut  Hyper vs Eut  Hypo vs Hyper | ANOVA  Bonferroni´s multiple comparison test | Table 2 | MAP values were similar between the three groups of animals |
| 13. LVIDd | LVIDd is altered in thyroid disorders | Heart  Eut rats  Hypo rats  Hyper rats | 5.38  6.02  4.3 | 0.46  0.46  0.46 | 15  15  15 | **p=0,025**  **p=0,020**  **p<0,001** | mm | Hypo vs Eut  Hyper vs Eut  Hypo vs Hyper | ANOVA  Bonferroni´s multiple comparison test | Table 2 | LVIDd increased and decreased in Hypo and Hyper rats, respectively |
| 14.LVIDs | LVIDs is altered in thyroid disorders | Heart  Eut rats  Hypo rats  Hyper rats | 2.70  3.12  2.10 | 0.15  0.39  0.27 | 15  15  15 | **p<0,001**  **p=0,023**  **p<0,001** | mm | Hypo vs Eut  Hyper vs Eut  Hypo vs Hyper | ANOVA  Bonferroni´s multiple comparison test | Table 2 | LVIDs increased and decreased in Hypo and Hyper rats, respectively |
| 15. AWTd | AWTd is altered in thyroid disorders | Heart  Eut rats  Hypo rats  Hyper rats | 1.50  1.35  1.55 | 0.08  0.04*  0.19 | 15  15  15 | **p=0,015**  p=ns  **p=0,020** | mm | Hypo vs Eut  Hyper vs Eut  Hypo vs Hyper | ANOVA  Bonferroni´s multiple comparison test | Table 2 | AWTd decreased in Hypo rats and did not change in Hyper animals. |
| 16. AWTs | AWTs is altered in thyroid disorders | Heart  Eut rats  Hypo rats  Hyper rats | 2.50  2.00  2.60 | 0.12  0.08*  0.19 | 15  15  15 | **p<0,001**  p=ns  **p<0,001** | mm | Hypo vs Eut  Hyper vs Eut  Hypo vs Hyper | ANOVA  Bonferroni´s multiple comparison test | Table 2 | AWTs decreased in Hypo rats and did not change in Hyper animals |
| 17. PWTd | PWTd is altered in thyroid disorders | Heart  Eut rats  Hypo rats  Hyper rats | 2.10  1.50  2.03 | 0.66  0.39*  0.58 | 15  15  15 | **p<0,001**  p=ns  **p<0,001** | mm | Hypo vs Eut  Hyper vs Eut  Hypo vs Hyper | ANOVA  Bonferroni´s multiple comparison test | Table 2 | PWTd decreased in Hypo rats and did not change in Hyper animals |
| 18. PWTs | PWTs is altered in thyroid disorders | Heart  Eut rats  Hypo rats  Hyper rats | 2.85  2.33  3.01 | 0.19  0.39*  0.19 | 15  15  15 | **p<0,001**  p=ns  **p<0,001** | mm | Hypo vs Eut  Hyper vs Eut  Hypo vs Hyper | ANOVA  Bonferroni´s multiple comparison test | Table 2 | PWTs decreased in Hypo rats and did not change in Hyper animals |
| 19.EF | EF is altered in thyroid disorders | Heart  Eut rats  Hypo rats  Hyper rats | 87  80  90 | 12  4  12 | 15  15  15 | **p<0,0001**  p=ns  **p<0,0001** | % | Hypo vs Eut  Hyper vs Eut  Hypo vs Hyper | ANOVA  Bonferroni´s multiple comparison test | Table 2 | EF decreased in Hypo rats and did not change in Hyper animals. |
| 20. FS | FS is altered in thyroid disorders | Heart  Eut rats  Hypo rats  Hyper rats | 55  45  57 | 12  8  4 | 15  15  15 | **p<0,0001**  p= ns  **p<0,0001** | % | Hypo vs Eut  Hyper vs Eut  Hypo vs Hyper | ANOVA  Bonferroni´s multiple comparison test | Table 2 | FS decreased in Hypo rats and did not change in Hyper animals |

NSE Thyroid-stimulating hormone (TSH); Triiodothyronine (T3); Thyroxin (T4); Body weight (BW); Heart rate (HR); Mean arterial pressure (MAP); LVID, left ventricle internal diameter; AWT, anterior wall thickness; PWT, posterior wall thickness; d, diastole; s, systole; EF, ejection fraction; FS, fractional shortening.
